# Recent Progress and Challenges on the Microfluidic Assay of Pathogenic Bacteria Using Biosensor Technology

**DOI:** 10.3390/biomimetics7040175

**Published:** 2022-10-25

**Authors:** Farnaz Bahavarnia, Mohammad Hasanzadeh, Deniz Sadighbayan, Farzad Seidi

**Affiliations:** 1 Jiangsu Co-Innovation Center for Efficient Processing and Utilization of Forest Resources and International Innovation Center for Forest Chemicals and Materials, Nanjing Forestry University, Nanjing 210037, China; 2 Pharmaceutical Analysis Research Center, Tabriz University of Medical Sciences, Tabriz 5166/15731, Iran; 3 Nutrition Research Center, Tabriz University of Medical Sciences, Tabriz 5166/15731, Iran; 4 Department of Biology, Faculty of Science, York University, Keel Street, Toronto, ON M3J 1P3, Canada

**Keywords:** microfluidic, pathogen, biomedical analysis, advanced nanomaterial, clinical infections

## Abstract

Microfluidic technology is one of the new technologies that has been able to take advantage of the specific properties of micro and nanoliters, and by reducing the costs and duration of tests, it has been widely used in research and treatment in biology and medicine. Different materials are often processed into miniaturized chips containing channels and chambers within the microscale range. This review (containing 117 references) demonstrates the significance and application of nanofluidic biosensing of various pathogenic bacteria. The microfluidic application devices integrated with bioreceptors and advanced nanomaterials, including hyperbranched nano-polymers, carbon-based nanomaterials, hydrogels, and noble metal, was also investigated. In the present review, microfluid methods for the sensitive and selective recognition of photogenic bacteria in various biological matrices are surveyed. Further, the advantages and limitations of recognition methods on the performance and efficiency of microfluidic-based biosensing of photogenic bacteria are critically investigated. Finally, the future perspectives, research opportunities, potential, and prospects on the diagnosis of disease related to pathogenic bacteria based on microfluidic analysis of photogenic bacteria are provided.

## 1. Introduction

Rapid screening and detection of pathogenic bacteria is a major challenge in the world. If pathogenic bacteria are not detected properly or on time, they can have irreversible effects. Bacterial diseases can generally be diagnosed with a delay in nonspecific clinical symptoms [1]. Due to the lack of care diagnostic methods, most clinical infections are misdiagnosed due to dependence on the patient’s symptoms rather than experimental tests. This leads to the administration of inappropriate drugs. Excessive use of antibiotics leads to resistance to bacterial infection [2]. Many cases of foodborne illness are reported worldwide each year (FDA, 2019), which is a constant necessity. Improving food safety emphasizes that it targets rapid, sensitive, specific, and cost-effective analytical methods for detecting microbial contamination worldwide. *Salmonella* is one of the major pathogenic bacteria worldwide. It is found in an exceedingly large variety of foods and causes diarrhea, gastroenteritis, typhoid, and other symptoms. *Salmonella* is estimated to cause 1.35 million infections annually, with 2600 hospitalizations and 420 deaths in U.S.A. The common recognition of such pathogens relies mainly on culture, ELISA, or PCR methods; however, these methods are considered simple and efficient; they are usually cumbersome, inaccurate, time-consuming, or require expensive and bulky equipment despite their high sensitivity. Therefore, the development of simple, rapid, and sensitive methods to detect pathogenic bacteria is an urgent request [3]. New methods need to be developed for the rapid and sensitive detection of bacteria. For example, various types of biosensors, including optical, electrochemical, piezoelectric, and colorimetric biosensors, have been used for the rapid detection of pathogens. Biosensors appear to be one of the most reliable analytical tools for detecting bacteria due to their advantages, such as being low-cost, label-free, simple, portable, and rapid. In recent years, microfluidic-integrated biosensors have played a crucial role in detecting pathogenic bacteria, especially for on-site screening, and lowering the risk of transmission. Microfluidics is a science that studies the technology of systems that process or manipulate smaller (10^−9^ to 10^−18^ L) amounts of liquid [4,5]. Polydimethylsiloxane (PDMS) is the most utilized material for fabricating microfluidic structures. It is optically transparent and capable of supporting important microfluidic components. Microfluidics enables the separate and cross-processing of multiple bond measurements with single or multiple samples simultaneously [6]. To identify single or multiple targets in a real or complex solution, the connection assay design usually involves more than one type of bioreceptors: the microfluidic structures, ensuring precise control of the experimental conditions [7]. Different factors, including flow velocity, sample volume, channel volume, channel height, reaction time, etc., can be precisely controlled by microfluidics structures [8]. Integration of biosensors with microfluidic channels enables reducing the sensing time by limiting the propagation distance between the sample molecules and the biosensor and creates a smooth flow on the biosensor for wide distribution of target molecules. Microfluidics provide a closed and stable environment to increase sensitivity [9]. By integrating microfluidic structures, sample processing and biosensing reactions are performed during a closed and comparatively stable environment, thus promising greater sensitivity and reliability [10]. In this review, we have explored the recent advances in pathogens evaluation microfluidic-integrated biosensors. We are going to not only consider the utilization of biosensors supported microfluidic technology in identifying pathogenic bacteria but also how to identify pathogens with old and new methods and a few future points of view.

**Scheme 1:**
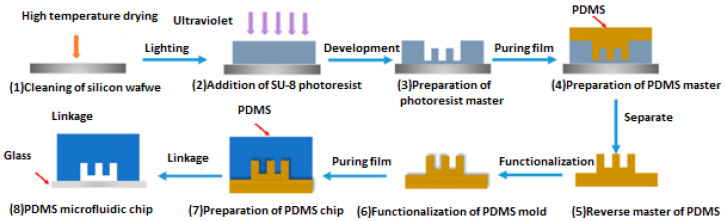
General procedure for the preparation of PDMS-based microfluidics.

Microfluidics could be a comparatively new field supported by a mix of biology, chemistry, physics, fluid dynamics, microelectronics, and materials science. Microfluidic technologies have become a strong tool in bioscience research laboratories over the past three decades. Further, the technology is promising for everyday applications because this sensor-based technology is used to detect pathogenic bacteria [11]. Several commercial devices are currently being tested to detect viruses, such as the immunodeficiency virus (HIV), coronavirus, herpes, and infectious hepatitis B and C. One of the essential steps in microfluidic usage is the optimal selection of materials for creating the device [12,13]. Because, at the micro-scale level, the properties are rather more enhanced, the platform material is probably going to affect the properties of synthesized nanomaterials. Particularly, unique phenomena occur in capillary microfluidics because of the shorter residence times, slow currents, increased heat transfer, and improved surface-to-volume ratio [14]. The most common polymers used to make microfluidic devices include polymethylsiloxane (PDMS), polymethyl methacrylate (PMMA), fluoropolymers, polymers and copolymers of cyclosporine (COPs/COCs), and thiols (TE) polymers [14,15]. These materials are effective for biology-related research, such as long-term cell screening, cell culture, and biochemical tests. PDMS-based devices can be used for detecting whole bacteria and proteins and their DNA, which offer great potential for detecting multiple targets [14,16]. For example, Scheme 1 exhibits the preparation process of PDMS microfluidics [17].

The mixing of biosensors with microfluidic systems offers an integrated and miniaturized alternative to the quality repetitive laboratory methods [18,19] because it offers an enormous reduction within the sample, reagent, energy consumption, and waste production [20,21]. The development of various technologies is additionally constantly transforming biological microfluidic chips to spice up their detection performance and broaden their application scenarios. Nanomaterials are often used for the surface immobilization and signal amplification of captured elements in microfluidic chips [22].

## 2. Current Methods of Bacterial Detection

Rapid diagnosis of pathogens is extremely important and necessary for the prohibition of diseases [23,24]. Here, we summarize a number of the broader cases of diagnostic techniques used to identify bacteria. Supervision is the prime control point in preventing diseases caused by pathogenic microorganisms. Efficient diagnostic methods are essential to managing this effect and have been executed over the years using conventional microbiological methods. These criterion methods have been a usual practice for nearly a century and are still a readily referenced practice for this detection type [25]. These customary methods rely almost exclusively on the applying of specific agar media lines to isolate, culture, and count viable cells in samples [26]. Some of the most current methods to confirm the presence of pathogens are typically supported by culture and colony counting methods and also the polymerase graft reaction (PCR) and enzyme-linked immunosorbent testing (ELISA). These methods take lots of time and are laborious to detect bacteria; however, the culture and enzyme-linked immunosorbent assay (ELISA), colony counting, and PCR methods take longer. Most of the methods applied for the detecting of bacteria are time-consuming and expensive [27].

Biosensors are widely employed in laboratories and industries because of their ease to use and low operational costs [23]. The biosensors are analytical tools that are generally used to detect specific elements. This method is easy, fast, and has high efficiency. Currently, these methods are widely (biosensors) applied for detecting pathogens due to their high sensitivity and selectivity [28]. We summarize a number of the foremost widely used techniques for detecting bacteria in Table 1.

As shown in Table 1, microchip-based biosensors offer better sensitivity and shorter response times in detection. While molecular detection is incredibly specific and sensitive, they always require pre-enrichment steps that take up most of the whole detection time.

## 3. Application of Microfluidic Biosensors for the Detection of Bacteria

**Scheme 2:**
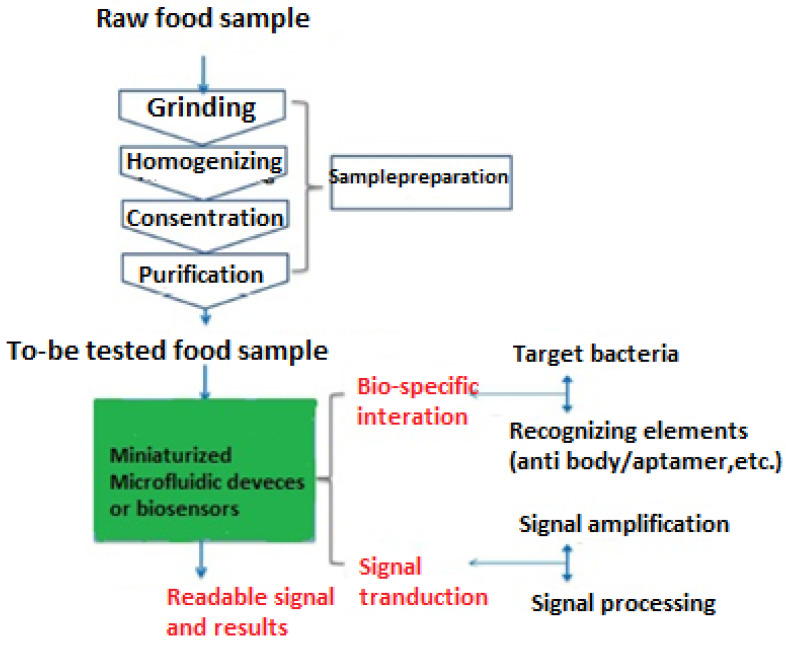
Chart of preparation and main steps involved in detecting pathogens by bio-sensing systems.

Microfluidics provide a closed and stable biosensing environment so to enhance sensitivity. For on-site portable biosensors, the effect of an open environment on sensing results significantly lowers the biosensor performance. By integrating the microfluidic structures, sample processing and biosensing reactions are carried out within a closed and comparatively stable environment, thus promising better sensitivity and reliability [37]. Microfluidic sensor systems are divided into different categories per the mechanism of their work. Supported by the transmission mechanisms, biosensors can be divided into optical biosensors (Raman scattering) [38,39,40,41,42,43], plasmonic resonance fiber grating (SPR) fiber [44,45,46,47], fluorescent [42,48,49,50,51,52,53], electrochemical biosensors [47,54,55,56,57,58,59], thermal biosensors, [60,61,62,63,64], and piezoelectric biosensors [65,66,67,68,69,70,71,72,73]. To view the specifics of the sample, several stages of sample preparation are required before the bioassay. For the identification of targets, the common pairs between targets and diagnostic elements include antibodies/antigens, enzymes/substrates, DNA or RNA/their complementary sequences, and aptamers or bacteriophages/whole bacterial cells. Readable electrochemical or optical signals are often transmitted through specific biological interactions by signal modules or external devices. Scheme 2, as an example, provides a quick overview of the detection of pathogens by bioassay devices. On the lower part, the current methods for bacteria detection support the integration of biosensors with microfluidic systems are summarized in different aspects.

### 3.1. Optical-Based Microfluidics Biosensor for the Detection of Pathogenic Bacteria

**Scheme 3:**
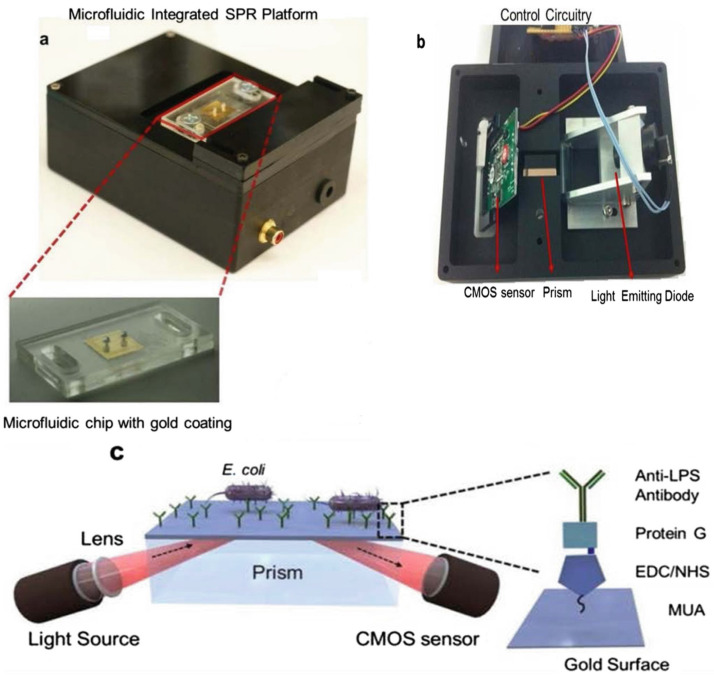
Portable plasmonic platform for pathogen detection and quantification. (**a**) The surface activated disposable microfluidic chips were mounted on the top side of the device. The microchip with the inlet and outlet ports, and the 50 nm thick gold coated glass substrate along with the disposable microchip is shown below. (**b**) The electronic setup of the device is represented from bottom. A light emitting diode illuminates a cylindrical lens, which collimates the light onto a rectangular prism. The reflected light is captured by a CMOS sensor, and the image is transferred to a portable computer using the control circuitry. The microfluidic chip is placed on the rectangular prism, with an refractive index matching oil in between. (**c**) Schematics of the microfluidic inte-grated SPR platform. The gold surfaces were modified with several activators (i.e., 11-Mercaptoundeconoic Acid (MUA), N-(3-Dimethylaminopropyl)–N9-ethylcarbodiimide Hydrochloride (EDC), N-Hydroxysuccinimide (NHS) and with antiLipopolysaccharide (LPS) an-tibody to capture the *E. coli*. The bacteria are captured by the antibodies in the microchannel, and the capture event induces a change in the local refractive index. This change provides a signature on the reflected light, which is captured by the sensor and transferred to a computerfor analysis.

The fluorescent-labeled method for detecting bacteria has good accuracy and high sensitivity. Additionally, the ability of the fluorescent labeling reagent directly determines the feasibility and sensitivity of the detection method. In recent years, fluorescent nanoparticles have attracted the eye of researchers because of their high intensity and low interference. Currently, some nanoscale fluorescent probes are wont to detect bacteria in microfluidic chips such as nano-gold, carbon dots (CD), and quantum dots (QD). Existing methods of optical detection on microfluidic chips include laser-induced fluorescence, chemiluminescence, immunofluorescence technique, and bioluminescence [71]. In another study, an in-situ pathogen detection system that supported the nano gene method for the definition and quantification of *E coli* O157:H7-specific gene was developed. By combining amino nano-magnetic grains with carboxyl quantum dots (QD 655), *E. coli* O157:H7 was measured by enriching chip sorting and fluorescence signal amplification detection, LOD detection limit (49 × 10^−15^). By combining a large range of microfluidic chips and fluorescence detectors, bacteria are often efficiently recognized with even more sensitivity. The benefits of fluorescent nanoparticles for detecting bacteria are their small size and significant number of atoms on the surface, high adsorption efficiency, and high surface reaction activity. The power to mix single bacteria with multiple nanoparticles at an identical time results in the upper fluorescence intensity of the required bacteria [72]. In another study, integration of a microfluidic system with SPRi has been developed to detect *Legionella pneumophila*; a tool equipped with an 800 nm semiconductor diode (nanometer) source, a tool camera (CCD), and a microfluidic cell was ready to detect the 16SrRNA sequence *Legionella peneumophila* with LOD = 0.45 FM [73]. Microfluidic technology combined with optical detection is extremely sensitive for the detection of low-concentration samples and has good prospects for the rapid detection of bacteria [37]. An integrated microfluid SPR (Scheme 3) for detection and determination of *E. coli* and *S. aureus* with LOD 3.2 × 10^7^ and 3.2 × 10^5^ CFU mL was reported in saline cluster phosphate salt solution with saline and peritoneal phosphate, respectively. During this system, specific antibodies are used to adsorb *E.coli* (anti-lipopolysaccharide/anti-LPS) and *S. aureus* (anti-lipoateic acid/anti-LAT) to activate disposable microfluidic chips with a gold plating surface.

Several activators, including 11-mercapto undecanoic acid (MUA), N-(3-dimethylaminopropyl)-N′-ethyl-carbodiimide hydrochloride (EDC), N-hydroxy succinimide (NHS) and G protein were used. When bacteria are trapped on the surface of a gold-plated microchannel, the signature of the reflected light is generated by a change within the local index of refraction, which is detected by a CMOS sensor and transmitted to a computer for analysis. All of the above systems are still under investigation and have not yet been commercialized. However, the results suggest that a conveyable, specific, and sensitive POC device is often developed for rapid on-site detection of pathogens. The mixing of SPR sensors in small chambers connected to multiple micro-channels in microfluidic systems can create a high-power SPR imaging sensor for multifunctional testing at different target locations [74].

In summary, the advantages of fluorescent nanoparticles have attracted the attention of researchers because of their high intensity and low interference. The benefits of fluorescent nanoparticles for detecting bacteria are their small size and a significant number of atoms on the surface, high adsorption efficiency, and high surface reaction activity. The fluorescence-based methods are restricted by the overlapping spectra of the target molecules and photobleaching, which significantly restricts their sensitivity and ability to analyze multiple molecules simultaneously. Quantum dots have numerous applications, especially in neuroscience [75] and oncology [76]. Nowadays, QDs have been increasingly utilized in bioanalysis as they can overcome the limitations of traditional fluorophores (low stability and photo bleaching).

Quantum dots are semiconducting nanocrystals with a cadmium selenium core and a zinc–sulfur shell [77]. These fairly new nanoparticles are often selected as fluorescent labels over organic dyes because they have a broad absorption spectrum, with the detection of a variety of various colored particles [78]. QDs-based fluorescence immunoassays are developed for the detection of veterinary drug residues, mycotoxins, and disease-related proteins; however, fluorescence-based techniques, as the primary method of detection, require light emission. The ECL (electrochemiluminescence) offers many advantages over fluorescence, attributed to the mechanism by which the excited state is produced. Despite fluorescence, ECL does not necessitate an external light source [79,80]; therefore, these properties make ECL suitable for integration into microfluidic devices. On the other hand, there are still some limitations of nanoparticle-based immunoassays. One of the most important problems to consider is their reproducibility. Fluorescence-based methods are also restricted by the overlapping spectra of the target molecules and photo bleaching, which significantly limits their sensitivity and ability to analyze multiple molecules simultaneously. Further, fluorescence-based measurements require expensive and sophisticated imaging tools [81].

### 3.2. Paper-Based Microfluidics Biosensors for Pathogen Detection

The paper-based microfluidic chips are a novel field because of their simple access, modification, processing, and disposal. Compared to traditional silicon, microfluidic and glass chips, silicon, microfluidic paper-based chips have unique advantages, including simplicity, affordability, low cost, high porosity, high physical absorption, simple manipulation and sterilization [82], and ability to work without equipment support [83]. Within the last ten years, microfluidic paper chip analysis technology has developed rapidly and has many practical perspectives on clinical diagnosis, food internal control, and environmental monitoring [84]. Cellulose is the main component of paper, which is a flexible material and has good biocompatibility. Biomolecules, such as enzymes, proteins, and DNA, are fixed on their surface, and therefore the white background of the paper incorporates a significant contrast. The background creates a color reaction. Additionally, the paper itself includes an excellent capillary effect and allows the liquid to pass passively through the capillary function of the paper microfluidic chip without the requirement for an external stimulus pump, while the reagents may be stored and transported. Recently, the detection of bacteria by microfluidic biosensors has attracted more attention because bacterial infection could be a major threat to animal and human health. The paper chip provides an analytical medium for detecting bacteria. Besides the features mentioned above, paper chips are often processed by incineration or natural destruction without contamination thanks to the environmentally compatible nature of cellulose. There are several diagnostic methods for paper-based microfluidic chip analysis, such as colorimetric, electrochemical, and optical detection. In one study, a colorimetric biosensor was developed for the detection of *Salmonella*. A nonspecific adsorption treatment and a simple approach to detect *Salmonella* were used, employing a microfluidic chip with a convergent and divergent helical structure and, therefore, the nano-mimetic enzyme MnO_2_ nanofibers (NFs) for rapid and sensitive detection of *Salmonella*. This biosensor can detect *Salmonella* from 4.4 × 10^1^ to 4.4 × 10^6^ CFU/mL in 45 min with a detection limit of 44 CFU/mL. MnO_2_ NFs are validated with good enzymatic activity, such as peroxidase and excellent environmental tolerance, resulting in increased sensitivity of this biosensor used for online and sensitive detection of *Salmonella*. This biosensor has been evaluated with high sensitivity, is useful for the diagnosis of *Salmonella*, and provides a promising platform for pathogens detection of pathogens through food [4]. In another study, an immune chromatography paper chip sensor was developed to simultaneously detect Pseudomonas aeruginosa and *Staphylococcus aureus*. Antibody-conjugated gold nanoparticles were used as signaling agents. The bacterial detection range was between 500 to 5000 CFU/mL. The advantage of a security disk sensor is that it does not require any biological sample preprocessing and is in a position to detect the whole part of the bacterial cell. Supported by the intensity of the dye, it converts the gold nanoparticles that accumulate within the test area to a voltage that is proportional to the bacterial concentration within the sample. The mix of a security disc and a transportable color reader provides a fast, sensitive, inexpensive, and quantitative tool for detecting an infective agent panel during a patient sample [85]. In one study, a fast paper-based, microphone-based, and smartphone-based test was developed to extract and directly detect fluorescent *Salmonella* typhimurium nucleic acids from the field and clinical specimens. The detection limit for *Salmonella typhimurium* in 10% poultry packing fluid with cellulose paper was 10^3^ CFU/mL, while that extracted with nitrocellulose paper was 10^4^ CFU/mL (as determined by PCR and fluorescence reflectance). Cellulose channels were more suitable for measuring low and extremely high concentrations of pathogen DNA, while nitrocellulose was better for analyzing intermediate concentrations. It was observed that DNA migrates through cellulose faster and sooner than cellulose due to charge–charge repulsion between nitrocellulose and DNA (both negatively charged), thus contributing to consistent and efficient extraction [86].

**Scheme 4:**
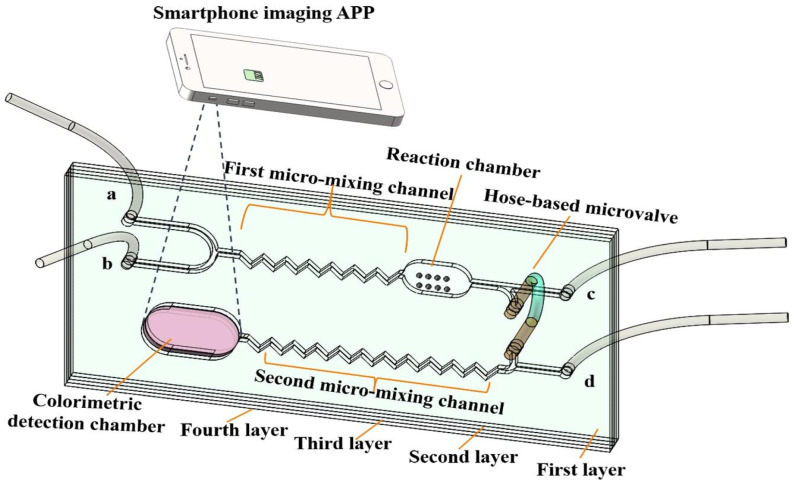
A graphical illustration of the microfluidic colorimetric biosensor developed using thiolated polystyrene microspheres for the detection of *Salmonella* typhimurium.

In one study, a microfluidic colorimetric biosensor was developed by ring-mediated isothermal amplification (LAMP) to detect monoplex pathogens. Six identical sets on a microfluidic compact disc (CD) were designed to perform 30 genetic analyzes of three different species of food pathogens. Sequential loading, mixing, and separation of LAMP primers/reagents and DNA sample solutions were performed using an optimal square wave microchannel, measuring chamber, and excretion control per minute (RPM). The 24 strains of pathogenic bacteria (*Vibrio cholerae*, *Escherichia coli*, and *Salmonella* spp.) were tested with eight strains of each bacterium and DNA amplification was performed on a microfluidic CD for 60 min. Calcine colorimetry was detected and analyzed by a smartphone. The system showed a detection limit (LOD) of 3 × 10^−5^ ng.μL^−1^ DNA by the color analysis testing chicken meat with three pathogenic bacteria [87]. In one study, a microfluidic colorimetric biosensor from polystyrene tubular microspheres (SH-PSs) for gold nanoparticle aggregation (AuNPs) and a smartphone-based imaging program to display colorimetric signals was developed for *Salmonella* detection. The aptamer-PS-cystamine conjugate was used as a diagnostic probe and reacted with *Salmonella* inside the samples. Compounds (cDNA-MNP) reacted with free aptamer-PS-cysteamine compounds as adsorption probes. Accumulation of AuNPs on the surface of *Salmonella*-aptamer-PS-cysteamine conjugates caused discoloration, which indicates different concentrations of *Salmonella*. LOD was 6.0 × 10^1^ CFU mL. Scheme 4 displays a graphical illustration of a microfluidic biometric sensor created using polystyrene microspheres to detect *Salmonella* [3].

The microfluidic paper-based chips have unique advantages, including simplicity, affordability, very low cost, high porosity, high physical absorption, simple manipulation and sterilization [88], and the ability to work without equipment support [89]; however, the developed method has some limitations. The applicability of the developed method needs to be established in different food matrices before its commercialization. In addition, as a limitation, the device’s suitability for multiplexing should be investigated to reduce the detection processing. Just a few of the developed devices have reached the market. Colorimetric detection is a sensitive and portable technique and is easily visible based on color change. Furthermore, the methods based on the colorimetric method are very simple and, in most cases, without the need for complex equipment, and with the naked eye, the color changes can be observed due to the reaction of nanomaterials with the desired analyte. Due to the fact that colorimetric methods are mostly based on the color change properties due to the accumulation of gold nanoparticles. On the other hand, the use of gold nanoparticles alone has less sensitivity and selectivity. Therefore, the use of DNA amplification methods can increase the sensitivity of the detection [55,89,90,91,92,93,94,95,96,97] and amplify the signal detection [94,98,99,100,101,102,103,104,105,106]. One of the disadvantages of colorimetric detection is the increase in the size of gold nanoparticles due to their accumulation in solution and, eventually, the formation of sediment; the color of the suspension becomes colorless over time and prevents accurate quantification. In addition, false-positive results due to the nonspecific accumulation of functional nanoparticles in complex biological samples limit their practical application.

### 3.3. Electrochemical-Based Microfluidics Biosensor for the Detection of Pathogenic Bacteria

Electrochemical biosensors are among the foremost common biosensors for bacteria detection due to their privileged merits of high sensitivity, low cost, and miniaturization potential. Supported different transducers are classified into amperometric, voltammetric, impedimetric, and potentiometric biosensors. Detection of bacteria by electrochemical-based microfluidics has the advantages of sensitivity and speed. When bacterial suspensions have the test hole under negative pressure, different pulse signals are generated due to the varied size and surface properties of the bacteria. After amplification and sorting, pulse signals will be converted into related information to the amount and sort of bacteria. In one study, a microfluidic colorimetric biosensor was developed by ring-mediated isothermal amplification (LAMP) to detect monoplex pathogens. Six identical sets on a microfluidic compact disc (CD) were designed to perform 30 genetic analyzes of three different species of food pathogens. Sequential loading, mixing, and separation of LAMP primers/reagents and DNA sample solutions were performed using an optimal square wave microchannel, measuring chamber, and excretion control per minute (RPM). The 24 strains of pathogenic bacteria (*Vibrio cholerae*, *E. coli*, and *Salmonella* spp.) were tested with eight strains of each bacterium, and DNA amplification was performed on a microfluidic CD for 60 min. Calcine colorimetry was detected and analyzed by a smartphone. The system showed a detection limit (LOD) of 3 × 10^−5^ ng.μL^−1^ DNA by the color analysis testing chicken meat with three pathogenic bacteria [81]. In one study, a microfluidic colorimetric biosensor based on polystyrene tubular microspheres (SH-PSs) for gold nanoparticle aggregation (AuNPs) and a smartphone-based imaging program to display colorimetric signals was developed for *Salmonella* detection. The aptamer-PS-cysteamine conjugate was used as a diagnostic probe and reacted with *Salmonella* inside the samples. Compounds (cDNA-MNP) reacted with free aptamer-PS-cystamine as adsorption probes. Accumulation of AuNPs on the surface of *Salmonella*-aptamer-PS-cystamine conjugates caused discoloration, which indicates different concentrations of *Salmonella*. LOD was 6.0× 10^1^ CFU/mL. Scheme 4 displays a graphical illustration of a microfluidic biometric sensor created using polystyrene microspheres to detection of *Salmonella* [3].

**Scheme 5:**
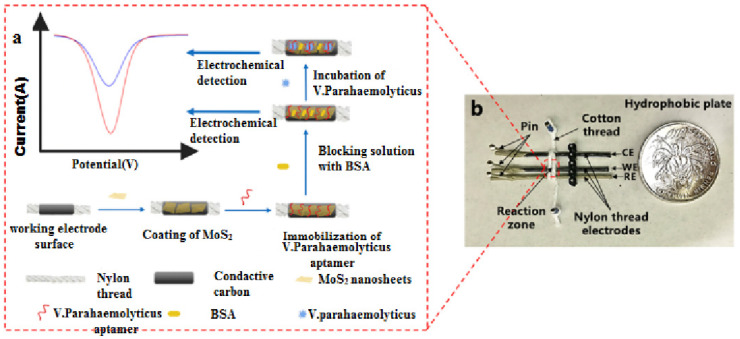
Illustration of detection mechanism of thread-based microfluidic aptasensor for *V. Parahaemolyticus* detection. (**a**) Schematic representation of detection mechanism of thread-based microfluidic aptasensor for *V. parahaemolyticus* detection. (**b**) Prototype of the final thread-based microfluidic ap-tasensor.

In one study, electrochemical aptasensor-based microfluidics was designed, manufactured, and tested using unlabeled immunogenic technology for rapid and sensitive detection of *V. parahaemolyticus* in seafood. Molybdenum disulfide nanosheets were commonly used to create more sensitivity in electrochemical measurements. The proposed aptasensor includes a detection limit of 5.74 CFU/mL and a dynamic detection range of 10–10^6^ CFU/mL. Compared to the quality method of page counting, the proposed aptasensor are used to provide higher detection sensitivity and a lower measurement time (30 min), which is mentioned in Scheme 5 [90].

In one study, an electrochemical-based microfluidic was developed for the detection of the Listeria bacteria. The method of identification of catalysts with microfluidics is fast and sensitive. By actively mixing the listeria cells, anti-listeria monoclonal antibodies modified the magnetic nanoparticles. Further, anti-Listeria polyclonal antibodies and urease-modified gold nanoparticles were incubated during a fluid separation chip. The apprehension efficacy of the Listeria cells within the separation chip was ~93% with a shorter time of 30 min because of the faster immune reaction using the active magnetic mixing. The changes on both impedance magnitude and point in time were demonstrated to be ready to detect the Listeria cells as low as 1.6 × 10^2^ CFU/mL. The detection time was reduced from the initial ~2 h to the present ~1 h [91]. An electrode-based multifaceted microfluidic chip for interactive loop-mediated isothermal amplification of macromolecule indium tin oxide for the detection of three important, urgent upper tract infections related bacteria, namely *Haemophilus influenza*, *Klebsiella pneumonia*, and mycobacteria were chosen for this study. Controlled the amplification process of measuring and analyzing the electrochemical signal of stain mycobacteria through eight-etched indium tin oxide electrochemical engineering. The results indicated that this assay, with the power to investigate multiplex genes qualitatively and quantitatively, is extremely specific, operationally simple, and expenditure/time effective. It exhibits high sensitivity with detection limits for mycobacterium tuberculosis with LOD 28 copies μL^−1^, *Haemophilus influenza* with LOD 17 copies μL^−1^, and *Klebsiella pneumonia*, respectively with LOD 16 copies μL^−1^. The entire differentiation is often finished during the brief time of 45 min, which has the potential to use in clinical diagnosis [92]. Another study used the mixed-color ring-assisted (LAMP) isothermal amplification method for the self-priming micro fluidization (SPC) chip, called on-chip mixing, which is a quick and quantitative method for *Vibrio parahemolyticus* detection. Color-based lamp (CMD-LAMP), compared to traditional methods, was as useful in the detection, reaching 1 × 10^3^ CFU/mL in contaminated food samples without pre-bacterial enrichment. Further, because of the employment of dye chips and SPC, the tiny result is often easily achieved by avoiding the requirement for complex tools and tedious operations. CMD-LAMP was also fast and cost-effective. At last, CMD-LAMP has great potential for performing quantitative on-site *Vibrio parahemolyticus* analysis for food safety [98].

Electrochemical biosensors have advantages such as simplicity, inexpensive, and quantitative detection. The μME-LAMP chip is disposable since both the PDMS chip and etched indium tin oxide (ITO) electrodes are easy to fabricate with high reproducibility and low cost. It also could be easily expanded to integrate as many micro chambers as possible and thus provides the possibility of ultra-high-throughput analysis of genes [87]; however, as a disadvantage, this method suffers from biosensor drift and surface effects on the electrodes after repeated exposure to biological or chemical materials, further reducing the sensitivity [99,100].

### 3.4. Immuno-Based Microfluidics Biosensor for Pathogen Detection

**Scheme 6:**
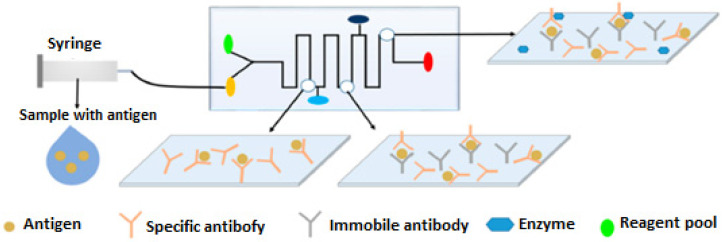
The fundamental principles of immune sensitivity-supported microfluidics for pathogen detection.

In recent years, point-of-view experiments have played a necessary role in safety assessment, biochemical analysis, and molecular detection, especially in low-resource environments. Immune sensitivity methods support the precise reaction between antigen and antibody for complex formation and offer high sensitivity, high specificity, and high analytical capacity. Among the assorted supervision point testing platforms, microfluidic chips have greater advantages. The microfluidic chip uses conventional laboratory shrinkage technology that permits the complete biochemical analysis process, including reagent loading, response, separation, and detection on the microchip. As shown in Scheme 6 immunological approaches to spot bacteria, hormones, parasites, viruses, proteins, various toxins, and other physiologically active substances, drug residues, and antibiotics [101]. Detection of pathogenic bacteria by immunosuppression strategy requires trained personnel because it is far more prone to infection than other methods. Nevertheless, when composed with microfluidic technology and security methods (Scheme 6), it can increase the specificity and sensitivity of the microfluidic analysis [102].

**Scheme 7:**
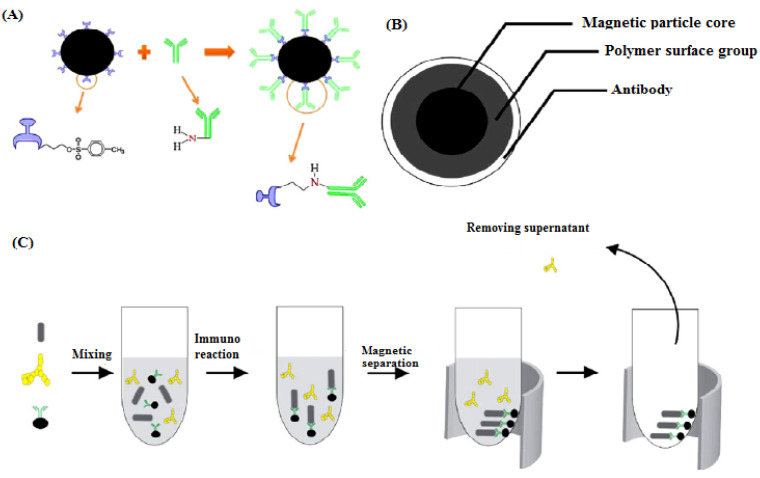
Images of the immune magnetic detachment process for the isolation of *Salmonella*. The illustration of IMB used in experiments. (**A**) The schematic mechanism of synthesizing IMB anti-*S. aureus*. (**B**) The structure of IMB. (**C**) Schematic illustration of the immunomagnetic separation process for separating *Salmonella* in a tube of the bioluminescence method. Scale bar = 10 m.

In another study, a mixture of thermal, chemical, and enzymatic lysis with magnetic bead-based DNA purification resulted in the best performance for Bacillus thuringiensis, which was used as a replacement for the bioarm and *Francisella tularensis* (*F. tularensis*) vaccine strains. Quantification of this bacterium was studied by standard safety methods and reinforced with nanomaterials within the concentration limited of 4 × 10^2^ GE/cm^2^ for *B. thuringiensis* and 4 × 10^3^ GE/cm^2^ for *F. tularensis*. LOD corresponded to 10^3^ (GE/mL) genome equivalents per ml for surface water samples with *F. tularensis* and 10^2^ GE/mL (genome equivalents per milliliter) for *B. thuringiensis*. In the air, 1 GE (genome equivalents) genome equivalents per milliliter genome equivalents per milliliter of *B. thuringiensis* per 10 L and 10 GE (genome equivalents) genome equivalents per milliliter genome equivalents per milliliter of *F. tularensis* at 10 L were scrutable. In these studies, to determine the appropriate approach for the chip-assisted procedure for DNA preparation, the use of real samples was demonstrated using livestock samples [107]. In one study, an easy and fast method for pathogen bacteria detection was developed using a microfluidic laboratory chip. The selectivity of the sensor was tested employing a two-way suspension of *Escherichia coli* and *Moraxella catarrhalis*. The detection limit of this microfluidic biosensor is now 9 × 10^5^ CFU/mL for *Escherichia coli* using multiple cell suspension perfusion. The chip sensibility with stabilized bacteria is managed by the peak of the sensing chamber, and ~10^4^ CFU/mL of *Escherichia coli* could easily be detected when a low-depth chamber (2 μm high) was employed. Development of more advanced lab chips with multiplex chambers and the possibility of different antibodies that allow concurrent detection of various bacteria strains are an inherent expansion of this work. The selectivity of the super sensor is demonstrated by testing the response with the mixture of bacterial spots, *E. coli*, and *M. catarrhalis*. By immobilizing different antibodies in parallel rooms, this approach can easily spread to a laboratory on a multi-chamber chip for the detection and identification of different bacteria during a complex solution [108]. In one study, a microfluidic chip with an immunofluorescence assay was developed to understand the sensitive and rapid isolation of diagnostic bacteria, which was applied to detect *Salmonella* in the presence of *Staphylococcus aureus* as an interfering agent. Bacterial concentrations were indirectly constant by detecting adenosine 5-triphosphate (ATP) using the bioluminescence BL reaction of firefly luciferine ATP; they found that BAC (Bacterial Artificial Chromosome) could emit sunlight when the BAC concentration was only 5.3×10^−2^% (*w*/*v*) (Weight/volume) and therefore the BL intensity reached its maximum at the concentration of BAC was 2.7 × 10^−2^% (*w*/*v*) (Weight/volume), which was 10-fold stronger than that without BAC. Supporting the origin of the IMB (immunomagnetic bead), a microfluidic chip composed of immunofluorescence approved for detachment and identifying bacteria concurrent was also developed. The target bacterial cells may be observed by a fluorescent microscope. This method had the benefits of short identification time, less sample utilization, good property and repeatability, and very sensitivity with up to 98% capture rate for target bacteria. In Scheme 7, images of the immunomagnetic detachment process for the seclusion of *Salmonella* in a tube by bioluminescence are shown [71].

In another study, immunoassay-based microfluidics was used to diagnose *Salmonella*-supported immune magnetic isolation, fluorescence labeling, and smartphone video processing. Originally, secure magnetic nanoparticles were utilized to effectively separate and concentrate target bacteria and magnetic bacteria. The magnetic bacteria were then labeled with immune fluorescent microspheres, and, therefore, the fluorescent bacteria were organized. Eventually, bacteria were injected sequentially into the microfluidic chip fluorescent on the smartphone, and therefore the difference between the frames was counted online, supporting the difference algorithm to detect the number of target bacteria. Compared to other strategies for the detection of *Salmonella*, the strategy of immune reaction within the tube and catalysis within the capillary showed higher sensitivity and better stability and was able to detect *Salmonella* up to 10^1^ CFU/mL in 2 h [109].

Detection of pathogenic bacteria by immunological methods requires trained personnel and alone is susceptible to cross-contamination and false-negative results. This is considered a limitation of the immunological method. However, when combined with microfluidic technology and immune methods, specific antigen-antibody reactions can increase the specificity and sensitivity of the microfluidic analysis. Additionally, the utilization of microfluidics is fast, low-consumption, and automatically compared to traditional immunology techniques. The most popular magnetic particles used in bioassays are iron oxides because they are easy to prepare and function and are considered biocompatible. Magnetic particles (MPs) disperse freely in the reaction mixture and, most importantly, can be easily isolated using an exterior magnet; therefore, solid carriers are ideals [85].

### 3.5. Aptamer-Based Microfluidics Biosensor for Pathogen Detection

Aptamers have drawn attention due to their unique ability to bind to an oversized range of non-nucleic acid targets with high affinity and specificity. Researchers are widely concentrated on aptamers as alternative promising bio-recognition ligands in food analysis within the past decades, particularly through their integration into microfluidic sensors for multi-analytes detection of very intricate food samples. Aptamers can easily be modified at their 5′ or 3′ terminals with thiols, amines, or epoxy groups to facilitate their immobilization in an exceedingly microfluidic chamber. In one study, a sensitive plasmonic-active biodevice was developed to detect *S. Typhimurium* bacteria. AuNPs (average diameter of 20 nm) were deposited uniformly on a transparent glass substrate, and the developed AuNPs-based plasmonic-active sensing chips were then conjugated successfully with aptamers by a simple dipping adsorption method. LSPR sensor chips showed high sensitivity and excellent selection, even within the presence of various background microorganisms. The developed chips were also able to detect quickly (within 30 to 35 min) with a detection limit above 1.0 × 10^4^ CFU/mL in pure culture, additionally to in artificially infected pork samples (without enrichment). Overall, this developed LSPR sensing chip has potential applications to detect hazardous pathogens in chemical and clinical laboratory settings, the agricultural industry, and also the food industry and environmental monitoring [110].

One study reported an exceedingly particular multiple methods to utilize aptamers because the molecular recognition elements, not to mention multicolor nanoparticles as luminescence labels for efficiently capturing and quantification of *S. aureus*, *V. parahemolyticus*, and *Salmonella* typhi in shrimp and milk samples with a limit detection of 25, 10, and 15 CFU/mL, respectively.

**Scheme 8:**
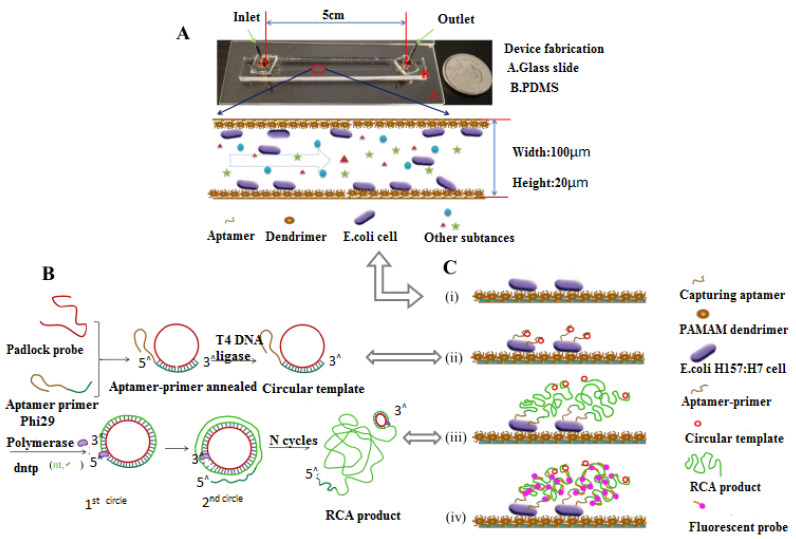
Designing of the dendrimer-aptamer-RCA detection system. Schematic drawing of the dendrimer-aptamer-RCA detection system: (**A**) image of the microfluidic device and illustration of target bacteria captured by dendrimer-aptamer modified PDMS detection system; (**B**) schematic representation of RCA based on a padlock probe-ligated circular template; (**C**) signal enhancement by RCA: (**i**) target bacterial cells are captured by the modified microchannel; (**ii**) aptamer-primer and padlock probe are sequentially introduced and circular template is formed via ligation; (**iii**) RCA is initiated and long tandem-repeat single strands are generated; (**iv**) fluorescent probes are added to visualize the detection signals.

Despite the variability of aptamer-based strategies extended up to now for food security analysis, aptamers make some restrictions, containing rapid destruction by nucleases, short duration of operation, the possibility of interaction with other components of samples, and reciprocal reaction with the target molecule, attention to all or any these restrictions is crucial for the event of an efficient detecting device [59]. In another study, a straightforward and sensitive microfluidic detection platform based on the supported fluorescence intensity was developed in which the PAMAM dendrimer was fixed on a PDMS microchannel and then has been modified with DNA aptamers to detect *E. coli* O157:H7 cells. To further improve the detection performance, RCA (Rolling circle amplification) was used to amplify the fluorescence signals. Additionally, RCA (Round Circuit Amplification) products were identified by agarose gel electrophoresis and atomic force microscopy. The results showed that RCA was able to increase the detection signals up to 50 times, and also, the detection limit of the system was reduced to 102 cells per milliliter with excellent detection characteristics; therefore, it is concluded that this straightforward RCA aptamer-based microfluidic system detection with enhanced RCA signal may be a hopeful method for the fast and sensitive identification of foodborne pathogens; therefore, it is expected that its simple design and intrinsic possibilities to integrate with other on-chip functionality modules, such as sample separation and concentration, will make this novel microchannel a promising tool for bacteria detection. Scheme 8 illustrates the design of the dendrimer-aptamer-RCA detection system [111].

In another study, a microfluidic system was developed that mixes both dendrimers -aptamers to identify *Escherichia coli* O157:H7. To attain this goal, 7th-generation polyamidoamine dendrimers were fixed on the detection surfaces of PDMS microfluid channels. The aptamers against *E. coli* O157:H7 were then conjugated on microchannel surfaces via stationary dendrimers as a template. Several analytical techniques, including FTIR (Fourier transform infrared), XPS spectra, water contact angles, fluorescence microscope, and AFM were used to confirm the successful surface modification in each step. The results demonstrated that the attractive approach considerably increased the number of aptamers in microfluidic channel surfaces to capture *E. coli* O157 cells. H7 was increased to permit sensitive detection, which successively led to the identification of *Escherichia coli* O157:H7 cells at low levels. The detection limit was around 10^2^ mL^−1^ cells. The results also showed that compared to the modified microchannels of 4-polyamidoamine (G4) dendrimers, those modified with G7 show improvement in detection signals, which can improve targeting absorption efficiency, and have higher throughput [112].

Aptamers have the potential as biorecognition elements for the selective capture of microorganisms. Aptamers can easily be modified at their 5′ or 3′ terminals with thiols, amines, or epoxy groups to facilitate their immobilization in an exceedingly microfluidic chamber; therefore, the APTA sensors combined with microfluidic technology can improve the sensitivity of detection of pathogenic microorganisms and realize the exactly automated real-time online testing, which can be prominent in future research; however, the microfluidic technology for constructing the APTA sensors to detect pathogenic microorganisms is seldom reported. Therefore, the APTA sensors combined with microfluidic technology can improve the sensitivity of detection of pathogenic microorganisms and realize the exact automated real-time online testing, which could be important in future research. Aptamers make some restrictions such as rapid destruction by nucleases, short duration of operation, the possibility of interaction with other components of samples, and reciprocal reaction with the target molecule; attention to all or some of these restrictions is crucial for the event of an efficient detecting device [113]. Finally, in Table 2, we summarize the microfluidic sensor-based methods used to identify pathogenic bacteria in this review article.

## 4. Conclusions and Future Perspectives

In this review, we have tried to highlight the latest findings in the field of pathogenic bacteria detection by microfluidic biosensors. The standard methods, such as ELISA and PCR-based techniques to detect bacteria, are time-consuming, costly, require specialized operators, and may lead to misleading results; therefore, it is essential to use methods that are not time-consuming and prone to contamination. In recent years, microfluidic-based biosensor systems, such as electrochemical-based microfluidic and optical-based microfluidic and immunoassay-based microfluidic and aptamer-based microfluidic, have been shown to be useful for the early detection of pathogens. The examples given by microfluidic biosensors for the detection of pathogenic bacteria show that rather simple samples consisting of pure cultures have been tested in a laboratory setting; we expect, with the advances made in this technology, it be used for samples that have complex components, such as various environmental samples and pharmaceutical products, identification of mutagens, and identification of toxins. In addition to the advent of microfluidic systems and the integration with these methods, the development of nanoparticles and nanomaterials has become an interesting topic. These materials provide a large specific surface area for chemical bonding. Such materials are used as a mobile substrate in biological and medical measurements and so on. Various materials are used for solid-phase affinity capture to provide an effective means for sample enrichment or signal enhancement. These materials include magnetic beads [114], agarose beads [115], and micro/nanostructures [57], among which magnetic nanomaterials (MPs) have a big advantage. Due to their magnetic properties, these materials can be manipulated independently of customary microfluidic or biological processes using permanent magnets or electromagnetic magnets. The additional degree of freedom caused by the relative motion of the magnetic beads with respect to the fluid causes the surface of the functionalized magnetic beads to be in the vicinity of the surrounding liquids and increases the concentration efficiency of the sample along with low-cost and great biocompatibility [116]. The microfluidic chip uses conventional laboratory shrinkage technology that permits the complete biochemical analysis process, including reagent loading, response, separation, and detection on the microchip. The use of nanomaterials in these biosensors will make these devices more sensible and practical in point-of-care early diagnosis. Early diagnosis will help to survive patients. Successful development of biosensors to diagnose various diseases will require finding the appropriate financing to move technology from research to commercial product realization. Given the advantages and disadvantages of the above methods, an ideal method should have several requirements: (1) it should be sensitive enough to be able to analyze very small amounts of the samples under study; (2) it should be of a specific size that manage to analyze several samples simultaneously and detect different types of bacteria; (3) must be able to detect bacteria without the need for expensive solutions or devices. The challenges of micro fluid technology include some liquid samples that are highly viscous and might be incompatible with the microfluidic device. However, the outlook is incredibly promising within the sphere of biosensors unified microfluidics may be referred to as:

- Microfluidic with high chemical and thermal stability for special applications.
- Integration of microfluidic and nano fluidic sensors for complete formation and application systems that do not require a specialized workforce operation.

It is hoped that in the future, with the advancement of science and technology and, therefore, the use of high-sensitivity nanomaterials and the elimination of the disadvantages of those methods, microfluidic methods will be used routinely to detect dangerous bacteria that affect human health and cause death.

## Figures and Tables

**Table 1:** Methods of bacteria detection along with detection limit and detection time.

Method	Bacteria	Limit of Detection (LOD)	Pre-Enrichment Time	Detection Time	Ref
Plate culture	*E. coli* O157:H7	10^5^ CFU/mL	48–72 h	Not reported	[29]
PCR	*E. coli* O157	1 CFU/mL	16–20 h	~3 h	[30]
PCR-ELISA	*E. coli*	10^2^ CFU/mL	16 h	~4 h	[31]
ELISA	*Shigella dysenteriae*	~10^4^ CFU/mL	48 h	~4 h	[32]
PCR-ELISA	*Salmonella* spp.	10^3^ CFU/mL	18 h	~5 h	[33]
Fiber optic biosensor	Listeria, *E. coli* O157:H7, *Salmonella*	~10^3^ CFU/mL ~10^3^ CFU/mL ~10^3^ CFU/mL	24 h 24 h 24 h	~2 h ~2 h ~2 h	[34]
Immune-based microchip	*E. coli*	50 CFU/mL	16 h	~3 h	[35]
Aptamer based biosensor	*E. coli* O157:H7	10 CFU/mL	16 h	~1.5 h	[36]

**Table 2:** Microfluidic sensors for detecting pathogenic bacteria (an overview).

Target Pathogen	Detection Limit	Method	Assay Time	Ref
*E. coli* O157:H7	49 × 10^−15^ CFU/mL	Surface plasmon resonance integrated microfluidic	Not reported	[72]
*Legionella pneumophila*	0.45 FM	Surface plasmon resonance integrated microfluidic	Less than 3 h	[73]
*E. coli* and *S. aureus*	3.2 × 10^7^, 3.2×10^5^ CFU/mL	Surface plasmon resonance integrated microfluidic	Not reported	[74]
*Salmonella*	4.4 × 10^1^ to 4.4 × 10^6^ CFU/mL	Microfluidic Colorimetric Biosensor	45 min	[4]
*Pseudomonas aeruginosa* and *Staphylococcus aureus*	500–5000 CFU/mL	Microfluidic Colorimetric Biosensor	Not reported	[85]
*Salmonella typhimurium*	10 ^3^ CFU/mL, 10 ^4^ CFU/mL	Paper microfluidic	Not reported	[86]
*Escherichia coli*, *Salmonella* and *Vibrio cholerae*	3 × 10^−5^ ng μL^−1^	microfluidic colorimetric biosensor supported by LAMP	60 min	[87]
*Salmonella*	6.0 × 10^1^ CFU/mL.	microfluidic colorimetric biosensor	Not reported	[3]
*Vibrio parahaemolyticus*	5.74 CFU/mL 10–10^6^ CFU/mL	electrochemical aptasensor -based microfluidic	30 min	[90]
*Listeria*	1.6×10^2^ CFU/mL	Electrochemical based microfluidic	~1 h	[91]
*Mycobacterium tuberculosis* (MTB), *Haemophilus influenza* (HIN), and *Klebsiella pneumonia* (KPN)	28 μL^−1^ 17 μL^−1^ 16 μL^−1^	electrode-based multiplex microfluidic chip supported by LAMP	45 min	[92]
*Vibrio parahemolyticus*	1 ×10^3^ CFU/mL	LAMP	Not reported	[98]
*Bacillus thuringiensis*, *Francisella tularensis*	4 × 10^2^ GE/cm^2^ 4 × 10^3^ GE/cm^2^	Immunoassay-based microfluidics biosensor	Not reported	[107]
*E. coli*, *M. catarrhalis*	9 × 10^5^ CFU/mL	Immunoassay-based microfluidics biosensor	Not reported	[108]
*Salmonella*	5.3 × 10^−2^% (*w*/*v*) by BAC 2.7 × 10^−2^% (*w*/*v*) without BAC. high sensitivity with up to 98%	immunofluorescence -based microfluidics	Not reported	[71]
*Salmonella*	10^1^ CFU/mL	immunoassay-based microfluidics	2 h	[109]
*S. typhimurium*	10 × 10^4^ CFU/mL	localized surface plasmon resonance (LSPR) sensing chip	within 30 to 35 min	[110]
*S. aureus*, *Vibrio parahemolyticus*, and *S. Typhimurium*	25 CFU/mL 10 CFU/mL 15 CFU/mL	multiplex lateral flow test strip sensor (Luminescence)	Not reported	[59]
*E. coli* O157:H7	10^2^ cells/mL	dendrimer-aptamer-RCA microfluidic	Not reported	[111]
*E. coli* O157:H7	10^2^ mL^−1^ cells	Mixes dendrimer-aptamer-RCA microfluidic	Not reported	[112]

## Data Availability

Not applicable.

## References

[1] Chuang T.-L., Chang C.-C., Chu-Su Y., Wei S.-C., Zhao X.-H., Hsueh P.-R., Lin C.-W. (2014). Disposable surface plasmon resonance aptasensor with membrane-based sample handling design for quantitative interferon-gamma detection. Lab Chip.

[2] Mairhofer J., Roppert K., Ertl P. (2009). Microfluidic systems for pathogen sensing: A review. Sensors.

[3] Man Y., Ban M., Li A., Jin X., Du Y., Pan L. (2021). A microfluidic colorimetric biosensor for in-field detection of *Salmonella* in fresh-cut vegetables using thiolated polystyrene microspheres, hose-based microvalve and smartphone imaging APP. Food Chem..

[4] Xue L., Jin N., Guo R., Wang S., Qi W., Liu Y., Li Y., Lin J. (2021). Microfluidic Colorimetric Biosensors Based on MnO2 Nanozymes and Convergence–Divergence Spiral Micromixers for Rapid and Sensitive Detection of *Salmonella*. ACS Sens..

[5] Luka G., Ahmadi A., Najjaran H., Alocilja E., DeRosa M., Wolthers K., Malki A., Aziz H., Althani A., Hoorfar M. (2015). Microfluidics integrated biosensors: A leading technology towards lab-on-a-chip and sensing applications. Sensors.

[6] Sarikaya A., Ladischi M., Geng T., Bhunia A., Apple H., Wereley S. (2001). Microfluidic Biocliip for Impedance Spectroscopy of Biological Species. Biomed. Microdevices.

[7] Whitesides G.M. (2006). The origins and the future of microfluidics. Nature.

[8] Foudeh A.M., Didar T.F., Veres T., Tabrizian M. (2012). Microfluidic designs and techniques using lab-on-a-chip devices for pathogen detection for point-of-care diagnostics. Lab Chip.

[9] Nasseri B., Soleimani N., Rabiee N., Kalbasi A., Karimi M., Hamblin M.R. (2018). Point-of-care microfluidic devices for pathogen detection. Biosens. Bioelectron..

[10] Sinha A., Tai T.-Y., Li K.-H., Gopinathan P., Chung Y.-D., Sarangadharan I., Ma H.-P., Huang P.-C., Shiesh S.-C., Wang Y.-L. (2019). An integrated microfluidic system with field-effect-transistor sensor arrays for detecting multiple cardiovascular biomarkers from clinical samples. Biosens. Bioelectron..

[11] Weng X., Neethirajan S. (2018). Paper-based microfluidic aptasensor for food safety. J. Food Saf..

[12] Yu Z., Tang Y., Cai G., Ren R., Tang D. (2019). Paper Electrode-Based Flexible Pressure Sensor for Point-of-Care Immunoassay with Digital Multimeter. Anal. Chem..

[13] Sachdeva S., Davis R.W., Saha A.K. (2021). Microfluidic point-of-care testing: Commercial landscape and future directions. Front. Bioeng. Biotechnol..

[14] Ren K., Zhou J., Wu H. (2013). Materials for microfluidic chip fabrication. Acc. Chem. Res..

[15] Pan L.-J., Tu J.-W., Ma H.-T., Yang Y.-J., Tian Z.-Q., Pang D.-W., Zhang Z.-L. (2018). Controllable synthesis of nanocrystals in droplet reactors. Lab Chip.

[16] Rivet C., Lee H., Hirsch A., Hamilton S., Lu H. (2011). Microfluidics for medical diagnostics and biosensors. Chem. Eng. Sci..

[17] Zhao X., Li M., Liu Y. (2019). Microfluidic-based approaches for foodborne pathogen detection. Microorganisms.

[18] Weibel D.B., Whitesides G.M. (2006). Applications of microfluidics in chemical biology. Curr. Opin. Chem. Biol..

[19] Hong J., Edel J.B., Demello A.J. (2009). Micro-and nanofluidic systems for high-throughput biological screening. Drug Discov. Today.

[20] Bringer M.R., Gerdts C.J., Song H., Tice J.D., Ismagilov R.F. (2004). Microfluidic systems for chemical kinetics that rely on chaotic mixing in droplets. Philos. Trans. R. Soc. London. Ser. A Math. Phys. Eng. Sci..

[21] Yager P., Edwards T., Fu E., Helton K., Nelson K., Tam M.R., Weigl B.H. (2006). Microfluidic diagnostic technologies for global public health. Nature.

[22] Choi S., Chae J. (2009). A regenerative biosensing surface in microfluidics using electrochemical desorption of short-chain self-assembled monolayer. Microfluid. Nanofluidics.

[23] Lazcka O., Del Campo F.J., Munoz F.X. (2007). Pathogen detection: A perspective of traditional methods and biosensors. Biosens. Bioelectron..

[24] Priyanka B., Patil R.K., Dwarakanath S. (2016). A review on detection methods used for foodborne pathogens. Indian J. Med. Res..

[25] Rohde A., Hammerl J.A., Appel B., Dieckmann R., Al Dahouk S. (2015). FISHing for bacteria in food–A promising tool for the reliable detection of pathogenic bacteria?. Food Microbiol..

[26] Houpikian P., Raoult D. (2002). Traditional and molecular techniques for the study of emerging bacterial diseases: One laboratory’s perspective. Emerg. Infect. Dis..

[27] Chen T.-L., Lee Y.-T., Kuo S.-C., Yang S.-P., Fung C.-P., Lee S.-D. (2014). Rapid identification of Acinetobacter baumannii, Acinetobacter nosocomialis and Acinetobacter pittii with a multiplex PCR assay. J. Med. Microbiol..

[28] Yao J., Yang M., Duan Y. (2014). Chemistry, biology, and medicine of fluorescent nanomaterials and related systems: New insights into biosensing, bioimaging, genomics, diagnostics, and therapy. Chem. Rev..

[29] Silk T.M., Donnelly C.W. (1997). Increased detection of acid-injured Escherichia coli O157:H7 in autoclaved apple cider by using nonselective repair on trypticase soy agar. J. Food Prot..

[30] Heijnen L., Medema G. (2006). Quantitative detection of *E. coli*, *E. coli* O157 and other shiga toxin producing *E. coli* in water samples using a culture method combined with real-time PCR. J. Water Health.

[31] Daly P., Collier T., Doyle S. (2002). PCR-ELISA detection of Escherichia coli in milk. Lett. Appl. Microbiol..

[32] Sapsford K.E., Rasooly A., Taitt C.R., Ligler F.S. (2004). Detection of Campylobacter and Shigella species in food samples using an array biosensor. Anal. Chem..

[33] Perelle S., Dilasser F., Malorny B., Grout J., Hoorfar J., Fach P. (2004). Comparison of PCR-ELISA and LightCycler real-time PCR assays for detecting *Salmonella* spp. in milk and meat samples. Mol. Cell. Probes.

[34] Ohk S.-H., Bhunia A.K. (2013). Multiplex fiber optic biosensor for detection of Listeria monocytogenes, Escherichia coli O157:H7 and *Salmonella* enterica from ready-to-eat meat samples. Food Microbiol..

[35] Wang S., Inci F., Chaunzwa T.L., Ramanujam A., Vasudevan A., Subramanian S., Ip A.C.F., Sridharan B., Gurkan U.A., Demirci U. (2012). Portable microfluidic chip for detection of Escherichia coli in produce and blood. Int. J. Nanomed..

[36] Wu W., Zhao S., Mao Y., Fang Z., Lu X., Zeng L. (2015). A sensitive lateral flow biosensor for Escherichia coli O157:H7 detection based on aptamer mediated strand displacement amplification. Anal. Chim. Acta.

[37] Kant K., Shahbazi M.-A., Dave V.P., Ngo T.A., Chidambara V.A., Than L.Q., Bang D.D., Wolff A. (2018). Microfluidic devices for sample preparation and rapid detection of foodborne pathogens. Biotechnol. Adv..

[38] Tunc I., Susapto H.H. (2020). Label-free detection of ovarian Cancer antigen CA125 by surface enhanced Raman scattering. J. Nanosci. Nanotechnol..

[39] Liu H., Li C., Song R., Xu S. (2019). A 3D mutilayer curved plasmonic coupling array with abundant and uniform hot spots for surface-enhanced Raman scattering. J. Phys. D Appl. Phys..

[40] Carneiro M.C., Sousa-Castillo A., Correa-Duarte M.A., Sales M.G.F. (2019). Dual biorecognition by combining molecularly-imprinted polymer and antibody in SERS detection. Application to carcinoembryonic antigen. Biosens. Bioelectron..

[41] Lee T., Mohammadniaei M., Zhang H., Yoon J., Choi H.K., Guo S., Guo P., Choi J.W. (2020). Single Functionalized pRNA/Gold Nanoparticle for Ultrasensitive MicroRNA Detection Using Electrochemical Surface-Enhanced Raman Spectroscopy. Adv. Sci..

[42] Li Z., Leustean L., Inci F., Zheng M., Demirci U., Wang S. (2019). Plasmonic-based platforms for diagnosis of infectious diseases at the point-of-care. Biotechnol. Adv..

[43] Sakir M., Salem S., Sanduvac S.T., Sahmetlioglu E., Sarp G., Onses M.S., Yilmaz E. (2020). Photocatalytic green fabrication of Au nanoparticles on ZnO nanorods modified membrane as flexible and photocatalytic active reusable SERS substrates. Colloids Surf. A Physicochem. Eng. Asp..

[44] Bhardwaj H., Sumana G., Marquette C.A. (2020). A label-free ultrasensitive microfluidic surface Plasmon resonance biosensor for Aflatoxin B1 detection using nanoparticles integrated gold chip. Food Chem..

[45] Das C.M., Ouyang Q., Dinh X.-Q., Coquet P., Yong K.-T. (2020). A theoretical insight into the use of anti-reflective coatings for the upliftment of sensitivity of surface plasmon resonance sensors. Opt. Commun..

[46] Farmani H., Farmani A., Biglari Z. (2020). A label-free graphene-based nanosensor using surface plasmon resonance for biomaterials detection. Phys. E Low-Dimens. Syst. Nanostruct..

[47] Zeng R., Gong H., Li Y., Li Y., Lin W., Tang D., Knopp D. (2022). CRISPR-Cas12a-Derived Photoelectrochemical Biosensor for Point-Of-Care Diagnosis of Nucleic Acid. Anal. Chem..

[48] Chen X., Nan Y., Ma X., Liu H., Liu W., Shi L., Guo T. (2019). In-situ detection of small biomolecule interactions using a plasmonic tilted fiber grating sensor. J. Lightwave Technol..

[49] Lao J., Han L., Wu Z., Zhang X., Huang Y., Tang Y., Guo T. (2019). Gold nanoparticle-functionalized surface plasmon resonance optical fiber biosensor: In situ detection of thrombin with 1 n·M detection limit. J. Lightwave Technol..

[50] Lobry M., Lahem D., Loyez M., Debliquy M., Chah K., David M., Caucheteur C. (2019). Non-enzymatic D-glucose plasmonic optical fiber grating biosensor. Biosens. Bioelectron..

[51] Loyez M., Ribaut C., Caucheteur C., Wattiez R. (2019). Functionalized gold electroless-plated optical fiber gratings for reliable surface biosensing. Sens. Actuators B Chem..

[52] Sypabekova M., Korganbayev S., González-Vila Á., Caucheteur C., Shaimerdenova M., Ayupova T., Bekmurzayeva A., Vangelista L., Tosi D. (2019). Functionalized etched tilted fiber Bragg grating aptasensor for label-free protein detection. Biosens. Bioelectron..

[53] He Y., Zhu L., Sun G., Meng F., Song Y. (2019). Fiber brag grating monitoring of a morphing wing based on a polyvinyl chloride reinforced silicone substrate. Opt. Fiber Technol..

[54] Zhou Q., Tang D. (2020). Recent advances in photoelectrochemical biosensors for analysis of mycotoxins in food. Trends Anal. Chem..

[55] Zhou Q., Lin Y., Zhang K., Li M., Tang D. (2018). Reduced graphene oxide/BiFeO3 nanohybrids-based signal-on photoelectrochemical sensing system for prostate-specific antigen detection coupling with magnetic microfluidic device. Biosens Bioelectron..

[56] Qiu Z., Shu J., Tang D. (2017). Bioresponsive Release System for Visual Fluorescence Detection of Carcinoembryonic Antigen from Mesoporous Silica Nanocontainers Mediated Optical Color on Quantum Dot-Enzyme-Impregnated Paper. Anal. Chem..

[57] Lv S., Tang Y., Zhang K., Tang D. (2018). Wet NH_3_-Triggered NH_2_-MIL-125(Ti) Structural Switch for Visible Fluorescence Immunoassay Impregnated on Paper. Anal. Chem..

[58] Yin Z., Zhu L., Lv Z., Li M., Tang D. (2021). Persistent luminescence nanorods-based autofluorescence-free biosensor for prostate-specific antigen detection. Talanta.

[59] Huang L., Wang J., Wang Q., Tang D., Yao L. (2020). Distance-dependent visual fluorescence immunoassay on CdTe quantum dot-impregnated paper through silver ion-exchange reaction. Microchim Acta..

[60] Huang L., Chen J., Yu Z., Tang D. (2020). Self-Powered Temperature Sensor with Seebeck Effect Transduction for Photothermal-Thermoelectric Coupled Immunoassay. Anal. Chem..

[61] Yu Z., Gong H., Xu J., Li Y., Xue F., Zeng Y., Liu X., Tang D. (2022). Liposome-Embedded Cu2- xAgxS Nanoparticle-Mediated Photothermal Immunoassay for Daily Monitoring of cTnI Protein Using a Portable Thermal Imager. Anal. Chem..

[62] Lv S., Zhang K., Zhu L., Tang D., Niessner R., Knopp D. (2019). H2-Based Electrochemical Biosensor with Pd Nanowires@ZIF-67 Molecular Sieve Bilayered Sensing Interface for Immunoassay. Anal. Chem..

[63] Azinheiro S., Kant K., Shahbazi M.A., Garrido-Maestu A., Prado M., Dieguez L. (2020). A smart microfluidic platform for rapid multiplexed detection of foodborne pathogens. Food Control.

[64] Zhang J., Liu Q., Ba Y., Cheng J., Yang H., Cui Y., Kong J., Zhang X. (2020). F-containing initiatior for ultrasensitive fluorescent detection of lung cancer DNA via atom transfer radical polymerization. Anal. Chim. Acta.

[65] Kim K.-I., Han C.-H., Choe Y.-A., Ju K.-S., Ri D.-H., Ri S.-B., Kim S. (2019). Influence of temperature and humidity on the detection of benzene vapor by a piezoelectric crystal sensor. Instrum. Sci. Technol..

[66] Pohanka M. (2019). Piezoelectric immunosensor for the determination of C-Reactive protein. Int. J. Electrochem. Sci.

[67] Urdinola K.B., Muñoz P.A.M., Marín P.A., Grajales M.J. (2019). In-Silico Prediction on the MSAMS-Assisted Immobilization of Bovine Serum Albumin on 10 MHz Piezoelectric Immunosensors. J. Mol. Eng. Mater..

[68] Yuan M., Zhang Q., Song Z., Ye T., Yu J., Cao H., Xu F. (2019). Piezoelectric arsenite aptasensor based on the use of a self-assembled mercaptoethylamine monolayer and gold nanoparticles. Microchim. Acta.

[69] Zamora-Sequeira R., Starbird-Pérez R., Rojas-Carillo O., Vargas-Villalobos S. (2019). What are the main sensor methods for quantifying pesticides in agricultural activities? A review. Molecules.

[70] Arora P., Sindhu A., Dilbaghi N., Chaudhury A. (2011). Biosensors as innovative tools for the detection of food borne pathogens. Biosens. Bioelectron..

[71] Qiu J., Zhou Y., Chen H., Lin J.-M. (2009). Immunomagnetic separation and rapid detection of bacteria using bioluminescence and microfluidics. Talanta.

[72] Mitchell K.A., Chua B., Son A. (2014). Development of first generation in-situ pathogen detection system (Gen1-IPDS) based on NanoGene assay for near real time *E. coli* O157:H7 detection. Biosens. Bioelectron..

[73] Foudeh A.M., Daoud J.T., Faucher S.P., Veres T., Tabrizian M. (2014). Sub-femtomole detection of 16s rRNA from *Legionella pneumophila* using surface plasmon resonance imaging. Biosens. Bioelectron..

[74] Tokel O., Yildiz U.H., Inci F., Durmus N.G., Ekiz O.O., Turker B., Cetin C., Rao S., Sridhar K., Natarajan N. (2015). Portable microfluidic integrated plasmonic platform for pathogen detection. Sci. Rep..

[75] Frasco M.F., Chaniotakis N. (2009). Semiconductor quantum dots in chemical sensors and biosensors. Sensors.

[76] SalmanOgli A. (2011). Nanobio applications of quantum dots in cancer: Imaging, sensing, and targeting. Cancer Nanotechnol..

[77] Zhang Z., Li Y., Li P., Zhang Q., Zhang W., Hu X., Ding X. (2014). Monoclonal antibody-quantum dots CdTe conjugate-based fluoroimmunoassay for the determination of aflatoxin B1 in peanuts. Food Chem..

[78] Sahoo S.K. (2022). Sensing and biosensing with optically active nanomaterials: A note. Sensing and Biosensing with Optically Active Nanomaterials.

[79] Fernández-Argüelles M., Costa-Fernández J.M., Pereiro R., Sanz-Medel A. (2008). Simple bio-conjugation of polymer-coated quantum dots with antibodies for fluorescence-based immunoassays. Analyst.

[80] Speranskaya E.S., Beloglazova N.V., Abé S., Aubert T., Smet P.F., Poelman D., Goryacheva I.Y., De Saeger S., Hens Z. (2014). Hydrophilic, bright CuInS2 quantum dots as Cd-free fluorescent labels in quantitative immunoassay. Langmuir.

[81] Reza K.K., Dey S., Wuethrich A., Wang J., Behren A., Antaw F., Wang Y., Sina A.A.I., Trau M. (2021). In situ single cell proteomics reveals circulating tumor cell heterogeneity during treatment. ACS Nano.

[82] Santhiago M., Nery E.W., Santos G.P., Kubota L.T. (2014). Microfluidic paper-based devices for bioanalytical applications. Bioanalysis.

[83] Noviana E., Ozer T., Carrell C.S., Link J.S., McMahon C., Jang I., Henry C.S. (2021). Microfluidic Paper-Based Analytical Devices: From Design to Applications. Chem. Rev..

[84] Martinez A.W., Phillips S.T., Butte M.J., Whitesides G.M. (2007). Patterned paper as a platform for inexpensive, low-volume, portable bioassays. Angew. Chem..

[85] Beveridge J.S., Stephens J.R., Williams M.E. (2011). The use of magnetic nanoparticles in analytical chemistry. Annu. Rev. Anal. Chem..

[86] Fronczek C.F., San Park T., Harshman D.K., Nicolini A.M., Yoon J.-Y. (2014). Paper microfluidic extraction and direct smartphone-based identification of pathogenic nucleic acids from field and clinical samples. RSC Adv..

[87] Sayad A., Ibrahim F., Uddin S.M., Cho J., Madou M., Thong K.L. (2018). A microdevice for rapid, monoplex and colorimetric detection of foodborne pathogens using a centrifugal microfluidic platform. Biosens. Bioelectron..

[88] Santhiago M., Kubota L.T. (2013). A new approach for paper-based analytical devices with electrochemical detection based on graphite pencil electrodes. Sens. Actuators B Chem..

[89] Carvalhal R.F., Simão Kfouri M., de Oliveira Piazetta M.H., Gobbi A.L., Kubota L.T. (2010). Electrochemical detection in a paper-based separation device. Anal. Chem..

[90] Jiang H., Sun Z., Guo Q., Weng X. (2021). Microfluidic thread-based electrochemical aptasensor for rapid detection of *Vibrio parahaemolyticus*. Biosens. Bioelectron..

[91] Chen Q., Wang D., Cai G., Xiong Y., Li Y., Wang M., Huo H., Lin J. (2016). Fast and sensitive detection of foodborne pathogen using electrochemical impedance analysis, urease catalysis and microfluidics. Biosens. Bioelectron..

[92] Luo J., Fang X., Ye D., Li H., Chen H., Zhang S., Kong J. (2014). A real-time microfluidic multiplex electrochemical loop-mediated isothermal amplification chip for differentiating bacteria. Biosens. Bioelectron..

[93] Liang C., Chu Y., Cheng S., Wu H., Kajiyama T., Kambara H., Zhou G. (2012). Multiplex loop-mediated isothermal amplification detection by sequence-based barcodes coupled with nicking endonuclease-mediated pyrosequencing. Anal. Chem..

[94] Zhang Y., Hu J., Zhang C.-Y. (2012). Sensitive detection of transcription factors by isothermal exponential amplification-based colorimetric assay. Anal. Chem..

[95] Zhao Y., Chen F., Wu Y., Dong Y., Fan C. (2013). Highly sensitive fluorescence assay of DNA methyltransferase activity via methylation-sensitive cleavage coupled with nicking enzyme-assisted signalamplification. Biosens. Bioelectron..

[96] Cui L., Ke G., Zhang W.Y., Yang C.J. (2011). A universal platform for sensitive and selective colorimetric DNA detection based on Exo III assisted signal amplification. Biosens. Bioelectron..

[97] Xu W., Xue X., Li T., Zeng H., Liu X. (2009). Ultrasensitive and selective colorimetric DNA detection by nicking endonuclease assisted nanoparticle amplification. Angew. Chem. Int. Ed..

[98] Pang B., Ding X., Wang G., Zhao C., Xu Y., Fu K., Sun J., Song X., Wu W., Liu Y. (2017). Rapid and quantitative detection of *Vibrio parahemolyticus* by the mixed-dye-based loop-mediated isothermal amplification assay on a self-priming compartmentalization microfluidic chip. J. Agric. Food Chem..

[99] Anik Ü. (2017). Electrochemical medical biosensors for POC applications. Medical Biosensors for Point of Care (POC) Applications.

[100] Sardesai N.P., Barron J.C., Rusling J.F. (2011). Carbon nanotube microwell array for sensitive electrochemiluminescent detection of cancer biomarker proteins. Anal. Chem..

[101] Wang X., Niessner R., Tang D., Knopp D. (2016). Nanoparticle-based immunosensors and immunoassays for aflatoxins. Anal. Chim. Acta.

[102] Zhu L., He J., Cao X., Huang K., Luo Y., Xu W. (2016). Development of a double-antibody sandwich ELISA for rapid detection of Bacillus Cereus in food. Sci. Rep..

[103] Dirks R.M., Pierce N.A. (2004). Triggered amplification by hybridization chain reaction. Proc. Natl. Acad. Sci. USA.

[104] Park C., Song Y., Jang K., Choi C.-H., Na S. (2018). Target switching catalytic hairpin assembly and gold nanoparticle colorimetric for EGFR mutant detection. Sens. Actuators B Chem..

[105] Li B., Jiang Y., Chen X., Ellington A.D. (2012). Probing spatial organization of DNA strands using enzyme-free hairpin assembly circuits. J. Am. Chem. Soc..

[106] Guo L., Xu Y., Ferhan A.R., Chen G., Kim D.-H. (2013). Oriented gold nanoparticle aggregation for colorimetric sensors with surprisingly high analytical figures of merit. J. Am. Chem. Soc..

[107] Julich S., Hotzel H., Gärtner C., Trouchet D., Ahmed M.F.E.M., Kemper N., Tomaso H. (2016). Evaluation of a microfluidic chip system for preparation of bacterial DNA from swabs, air, and surface water samples. Biologicals.

[108] Boehm D.A., Gottlieb P., Hua S.Z. Surface functionalization of a microfluidic biosensor for bacteria detection and identification. Proceedings of the Sensors and Smart Structures Technologies for Civil, Mechanical, and Aerospace Systems 2007.

[109] Huang F., Xue L., Qi W., Cai G., Liu Y., Lin J. (2021). An ultrasensitive impedance biosensor for *Salmonella* detection based on rotating high gradient magnetic separation and cascade reaction signal amplification. Biosens. Bioelectron..

[110] Oh S.Y., Heo N.S., Shukla S., Cho H.-J., Vilian A., Kim J., Lee S.Y., Han Y.-K., Yoo S.M., Huh Y.S. (2017). Development of gold nanoparticle-aptamer-based LSPR sensing chips for the rapid detection of *Salmonella* typhimurium in pork meat. Sci. Rep..

[111] Jiang Y., Zou S., Cao X. (2017). A simple dendrimer-aptamer based microfluidic platform for *E. coli* O157:H7 detection and signal intensification by rolling circle amplification. Sens. Actuators B Chem..

[112] Hao X., Yeh P., Qin Y., Jiang Y., Qiu Z., Li S., Le T., Cao X. (2019). Aptamer surface functionalization of microfluidic devices using dendrimers as multi-handled templates and its application in sensitive detections of foodborne pathogenic bacteria. Anal. Chim. Acta.

[113] Macedo B., Cordeiro Y. (2017). Unraveling prion protein interactions with aptamers and other PrP-binding nucleic acids. Int. J. Mol. Sci..

[114] Cai H., Parks J., Wall T., Stott M., Stambaugh A., Alfson K., Griffiths A., Mathies R., Carrion R., Patterson J. (2015). Optofluidic analysis system for amplification-free, direct detection of Ebola infection. Sci. Rep..

[115] Lee J., Kim H., Heo Y., Yoo Y.K., Han S.I., Kim C., Hur D., Kim H., Kang J.Y., Lee J.H. (2020). Enhanced paper-based ELISA for simultaneous EVs/exosome isolation and detection using streptavidin agarose-based immobilization. Analyst.

[116] Gao L., Zhuang J., Nie L., Zhang J., Zhang Y., Gu N., Wang T., Feng J., Yang D., Perrett S. (2007). Intrinsic peroxidase-like activity of ferromagnetic nanoparticles. Nat. Nanotechnol..

